# A multi-scale approach to detecting standing dead trees in UAV RGB images based on improved faster R-CNN

**DOI:** 10.1371/journal.pone.0281084

**Published:** 2023-02-24

**Authors:** Xiangtao Jiang, Zhenyu Wu, Siyu Han, Hui Yan, Bo Zhou, Jianjun Li

**Affiliations:** 1 College of Computer Science and Information Technology, Central South University of Forestry and Technology, Changsha, China; 2 Hunan Linkeda Information Science and Technology Co.LTD, Changsha, China; Universidade Federal de Uberlandia, BRAZIL

## Abstract

The health of the trees in the forest affects the ecological environment, so timely detection of Standing Dead Trees (SDTs) plays an important role in forest management. However, due to the large spatial scope of forests, it is difficult to find SDTs through conventional approaches such as field inventories. In recent years, the development of deep learning and Unmanned Aerial Vehicle (UAV) has provided technical support for low-cost real-time monitoring of SDTs, but the inability to fully utilize global features and the difficulty of small-scale SDTs detection have brought challenges to the detection of SDTs in visible light images. Therefore, this paper proposes a multi-scale attention mechanism detection method for identifying SDTs in UAV RGB images. This method takes Faster-RCNN as the basic framework and uses Swin-Transformer as the backbone network for feature extraction, which can effectively obtain global information. Then, features of different scales are extracted through the feature pyramid structure and feature balance enhancement module. Finally, dynamic training is used to improve the quality of the model. The experimental results show that the algorithm proposed in this paper can effectively identify the SDTs in the visible light image of the UAV with an accuracy of 95.9%. This method of SDTs identification can not only improve the efficiency of SDTs exploration, but also help relevant departments to explore other forest species in the future.

## 1. Introduction

As an essential part of the forest, trees are essential for maintaining the balance of the ecosystem [1, 2]. During their long growing process, trees are affected by several factors, either individually or together, including trees themselves, intraspecific competition, pests and diseases, human activities, and natural disasters. Some or all of the trees die but do not fall, thus becoming Standing Dead Trees (SDTs) [3, 4], also known as snags. The formation of SDTs is an important ecological process [5], and as a source of forest carbon stocks [6], SDTs are vital for the sustainable development of forests [7]. Timely detection of SDTs and identification of their spatial distribution is the basis for studying the ecological mechanism of tree death. At the same time, it also assists the forestry department with the scientific management of forests [8].

Identifying SDTs in forests has been challenged by wide spatial distribution and strong randomness [9], so the process has always been complicated and tedious. Limited by technology and equipment in the early times, information on STDs was primarily collected through manual field surveys [10]. Given the random sampling method used in field surveys, the accuracy of the overall survey was related to the selection of samples to a large extent, and it was time-consuming and labor-intensive [11]. Therefore, a method for identifying aerial and satellite remote sensing images [12–14] has been gradually developed. The method mainly identifies remote sensing images based on the observer’s knowledge, experience, and skills [15]. Early equipment had lower resolution and could not obtain the precise location of standing dead trees, so the distribution of SDTs in a certain area could only be roughly judged. However, because satellites acquire images in spatially contiguous regions, and therefore, large-scale research can be carried out, which effectively promotes research on the mechanism of tree death [16]. Because the decrease in chlorophyll absorption in SDTs results in a significant difference between the increase in red reflectance and the decrease in Near Infrared (NIR) reflectance in the spectrograms acquired by satellites [17]. Given this feature, using Normalized Difference Vegetation Index (NDVI) and Red-Green Index (RGI)to identify SDTs has also proved effective [18, 19]. But this type of method is easily affected by similar spectra and could lead to large errors. Comparatively speaking, these early methods are unable to identify SDTs efficiently and accurately, which can hinder the efforts of forestry departments to work effectively.

Considering the limitations of the methods above, machine learning methods were introduced to detect SDTs [20]. Early machine learning needs manual construction of computational factors and extracted target features based on the Scale-Invariant Feature Transform (SIFT) [21] and the Histogram of Oriented Gradient (HOG) [22]. Target features were subsequently classified by methods such as the Support Vector Machine (SVM), the Random Forest (RF), and the K-nearest Neighbors (K-NN). In so doing, object detection was completed with bounding box correction. Researchers have found that texture features can be more effective in identifying SDTs than previous methods using only spectral characteristics [23]. Also, the validates the effectiveness of machine learning in identifying SDTs [24].

Recently, limited by the satellite revisit period, it has been difficult to obtain high-precision and high-efficiency SDTs data. Unmanned Area Vehicles (UAVs) provide supplementary methods because of their small size, good maneuverability, real-time performance, and a variety of imaging sensors that can be carried [25]. In particular, UAVs are equipped with a GPS, which can achieve higher position accuracy for any object. Existing research focuses on obtaining data with the use of multi-spectral, lidar, and common three-channel sensors in a single or combined manner. It has been proved that Random Forests (RF) and Support Vector Machines with Polynomial Kernel (SVMP) algorithms can effectively identify SDTs in multispectral images with relatively high accuracy and short processing time [24]. As laser beams of LiDAR can penetrate forest canopies, the three-dimensional structural features of the tree canopy can be effectively obtained and accurate segmentation. Thus, it has been found that tree health status detection can be performed on laser data by SVM [26]. The digital cameras carried by UAVs account for the largest proportion of all sensors, but only a few researchers proposed using RGB images obtained by digital cameras to identify SDTs. Existing identification methods include visual interpretation, HSV thresholding, the method of combining spectral with texture analyses, and a super-pixel segmentation algorithm, also called linear spectral clustering for SVM [27–29]. Though traditional object detection methods can effectively identify SDTs in different types of images, given the different characteristics of SDTs of different species, such methods need to first calculate extraction factors based on artificially designed features, which leads to poor robustness.

In recent years, with the rapid development of machine learning, object detection algorithms based on convolutional networks have emerged. Unlike traditional methods that generally use artificially designed feature operators to identify images, the current object detection algorithms can independently extract deep features of the target and acquire information about the shape, texture, and spatial relationships of images. Since more semantic information can be obtained in the image, it can achieve better detection results than traditional methods [30, 31]. According to whether there are candidate frames, object detection algorithms based on deep learning can be divided into two categories, single-stage algorithms like YOLO, and two-stage algorithms represented by the RCNN series. Both have their advantages, the one-stage algorithms are faster, whereas the two-stage ones yield better accuracy. Since convolutional networks have apparent advantages over traditional methods, they have been used in many remote sensing scenes, including ship detection, house detection, land classification, etc. [32, 33]. For the use of convolutional networks to identify SDTs in remote sensing images, some researchers have conducted research, such as changing the combination of the number of spectral channels and resolution to compare the effect of SDTs recognition [34], or using three-dimensional deep neural networks to identify SDTs on multi-source imagery with radar and multispectral fusion [35, 36]. There are also researchers using multi-scale methods to identify SDTs in multi-spectral images captured by drones [37].

The Convolutional networks have overcome the shortcomings of traditional methods, but existing research mainly focuses on using convolutional networks to identify SDTs in multi-source data, and rarely on a single data source, like RGB images captured by digital cameras on UAVs. This is due to the wide-scale span, resulting in poor performance in recognizing small targets. Moreover, RGB images have less exploitable information than multi-source images. Both of these pose challenges for identifying SDTs in ordinary RGB images. Most UAVs tend to be equipped with digital cameras to capture images, so it is necessary to enhance their recognition rate of identifying SDTs in RGB images. It has to be pointed out that although convolutional networks can extract features from remote sensing data, they only focus on local information, and with the absence of global information, they cannot acquire all contextual information. Some researchers have found that Transformer’s self-attention mechanism [38] can process long-distance dependencies and perform parallel computing without being constrained by local interactions, which can achieve good results in object detection. To solve the scale problem of SDTs in visible light images obtained by UAVs in convolutional networks and the problem that convolutional networks only focus on nearby pixels but not features at a long distance, this paper proposes a method integrating Transformer and multi-scale structure. This method can construct global information from the entire image and fully learn contextual information. In this way, texture and color features can be fully utilized to identify SDTs. This paper makes the following contributions:

1. Given the potential problems of imaging equipment in actual operations, we use UAVs with digital cameras to obtain RGB images, then generate an SDTs dataset for experimentation.
2. In order to increase the degree of automation, we use the convolutional network together with the Transformer to reduce human effort by autonomous learning of features from global information.
3. Regarding the difficulty of multi-scale object detection caused by a single data source within UAV visible light images, a multi-scale feature balance enhancement module is used to improve the multi-scale recognition accuracy with SDTs, especially small ones.
4. The rest of the paper is as follows. Section 2 illustrates the study area and obtained data while Section 3 introduces the proposed methods. In Section 4, experimental comparisons are made, followed by a detailed analysis. The last section concludes the paper.

## 2. Materials

### 2.1. Study area

**Fig 1:**
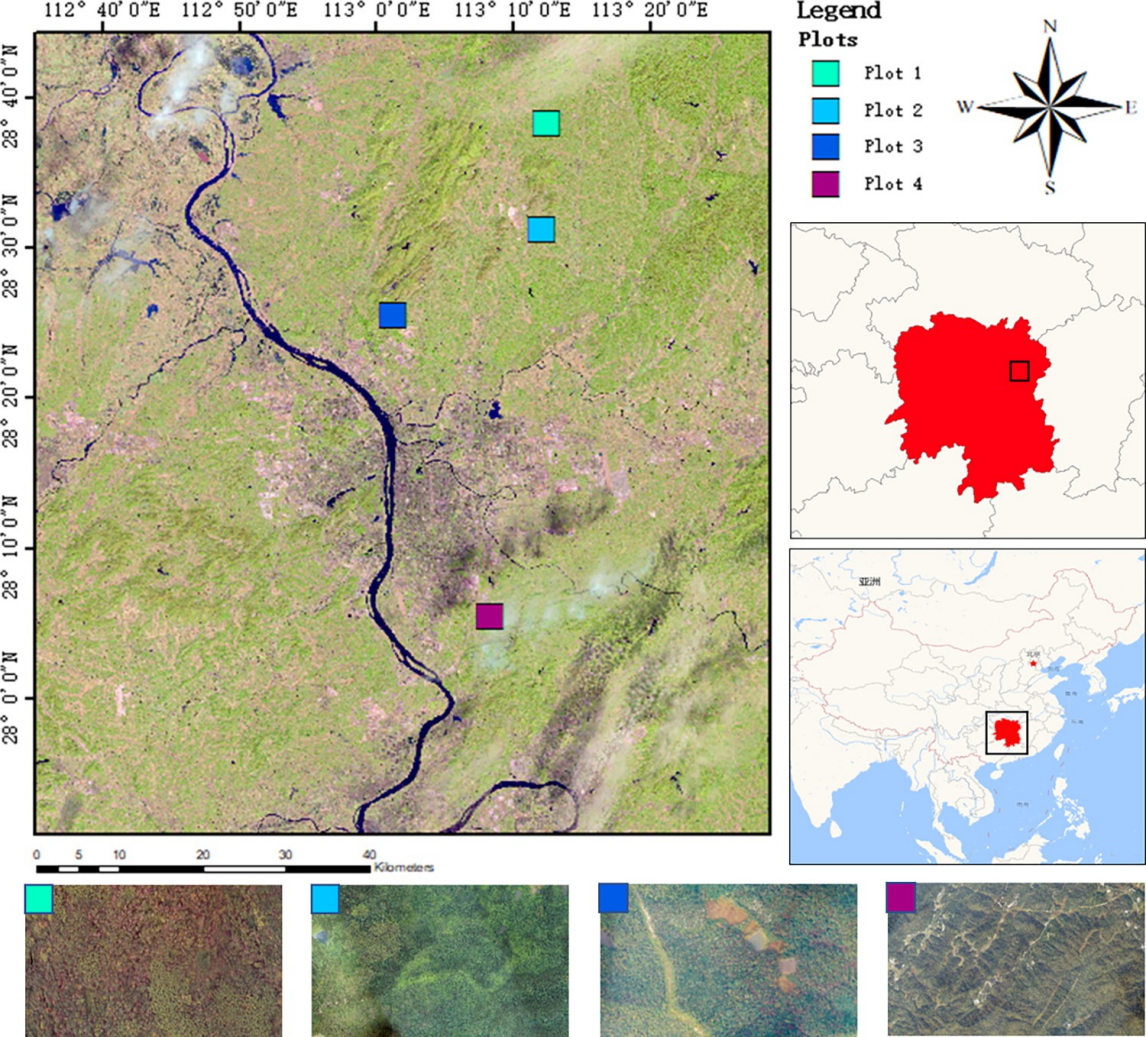
Study area and experimental area (The base map for the top half comes from USGS (www.usgs.gov)).

The study area is located in Changsha County, Changsha City, Hunan Province in the People’s Republic of China, as shown in Fig 1. The area lies between the geographical coordinates of 112°56′15″E, 27°54′55″N and the geographical coordinates of 113°36′00″E, 28°38′55″N. It has a humid continental and subtropical monsoon climate, with four distinct seasons characterized by a short cold winter and a long hot summer. The dense vegetation coverage is a good data source for research.

### 2.2. Data acquisition and preprocessing

The present study chooses the four sampling areas shown at the bottom of Fig 1 as sites for data collection. The DJI Matrice M300 RTK was used for data collection, which is equipped with a DJI Zenmuse H20T multi-function digital camera and a gimbal. The study only adopts a wide-angle lens for image acquisition. Specific information for the equipment is shown in Table 1 (Information provided by DJI). On sunny and breezy days in September 2021, we used the "DJI pilot" application to plan the route and set the interval of aerial photography. Given the differences in terrain types, the Matrice M300 RTK flew with a constant speed of 8m/s at a height of 150m. The camera lens is shot vertically downward, the frontal overlap rate was 75% and the side overlap rate was 70%. After the UAV completes the shooting of the visible light image of the entire test area, we would use the ‘PhotoScan’ software to stitch the images, generating a Digital Orthophoto Map (DOM) of the entire test area. Meanwhile, the DOM was geometrically corrected by a ground GPS, which facilitates the production of subsequent data sets.

**Table 1:** Parameters for data acquisition equipment.

Name	Parameters
**DJI MatriceM300 RTK**	**Dimensions:** 430×420×430 mm **Weight:** 6.3KG **Max Takeoff Weight:** 9 kg **Max Wind Resistance:** 15m/s **Max Speed:** 23m/s **Max Flight Time:** 55min
**DJI Zenmuse H20T**	**Sensor:** 1/2.3" CMOS,12 MP **Video:** 1920×1080@30fps **Photo Size:** 4056 x 3040

### 2.3. SDTs dataset

**Fig 2:**
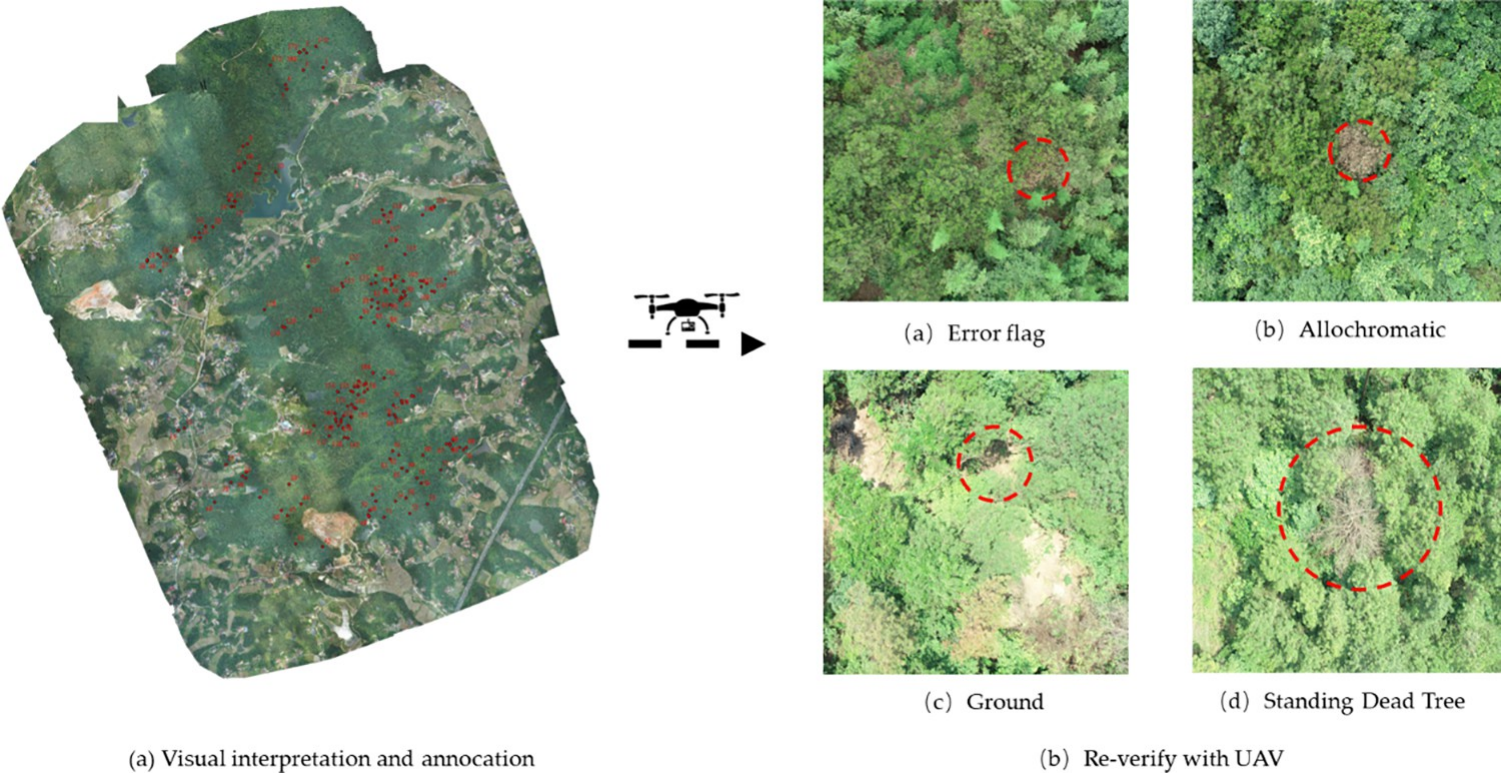
Manually mark suspected points and Re-verify with UAV.

Since data sets of SDTs are not common and no public data sets are available, our study uses based on the orthorectified images generated from the visible images of the four test areas acquired by the UAV. The ‘Gdal’ library in Python is used to automatically crop the TIF format DOM images of the four test areas to generate images that can be used for marking. Considering the limitations of the computer hardware, and in order to allow the network to better understand SDTs features in the sample data, the images are cropped to a resolution of 512x512 pixels, and images in JPEG format are selected as the experimental sample. A total of 52,882 images are cropped, and the center coordinate position of each image is preserved. A forestry worker with more than ten years of experience in charge of marking SDTs (as shown in Fig 2(A), which is shown in the No. 2 test area as an example, after visual interpretation, the standing dead trees are marked on the original orthophoto image) and interpreting the cropped images. Then the UAV is used for Reverification and screening with the marked longitude and latitude coordinates. As errors are inevitable in GPS positioning, we selected the distance of 5m near the marked points as the margin of error. Fig 2(B) refers to the visual interpretation of the marked points during flight verification.

**Fig 3:**
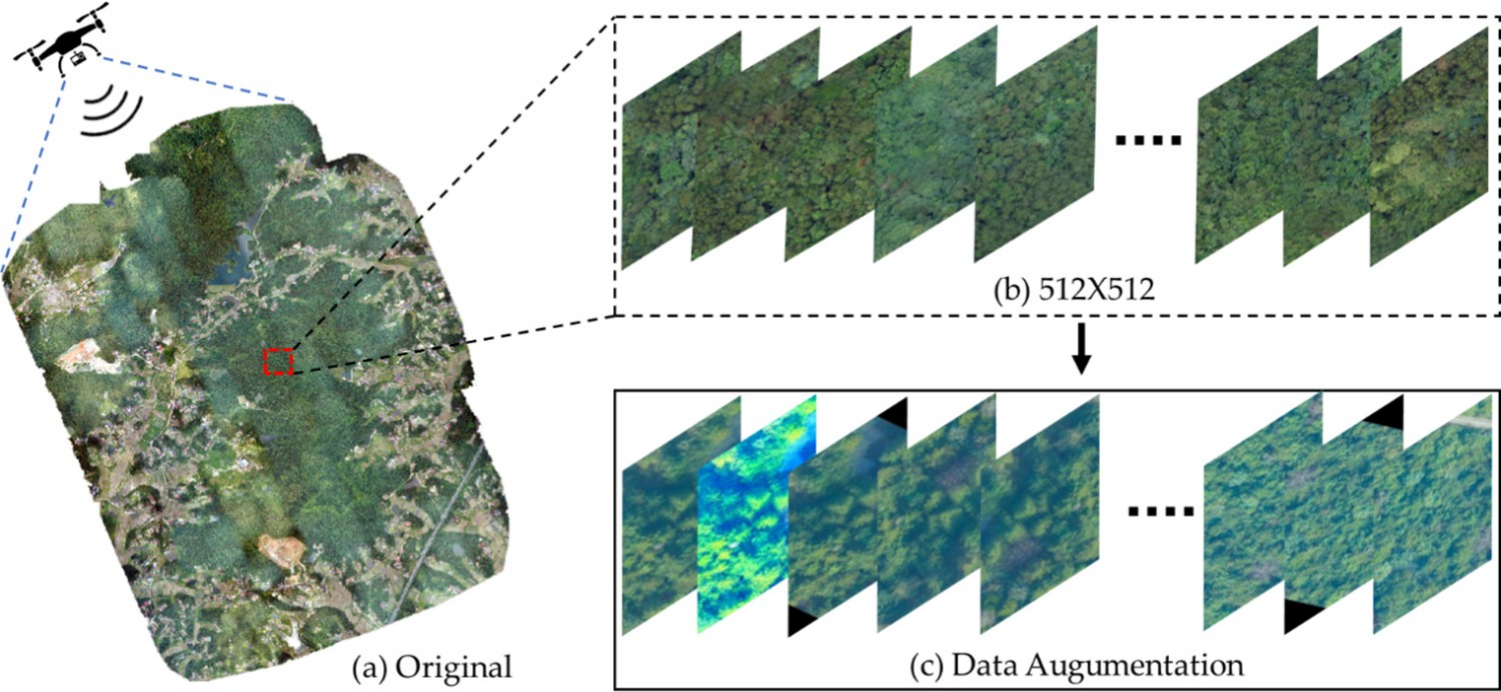
**SDT dataset production.** (a) Orthophoto images obtained by UAV; (b) Crop the original image to 512*512 size; (c) Data Augmentation.

The proportion of SDTs in the forest is relatively small. As shown in Table 2, a total of 568 pictures representing 978 SDTs are finally finalized after an on-site inspection. Obviously, such a sample size is small, which may result in a lack of study data. Consequently, relevant features may not be acquired, which will influence the experimental results. Studies have shown that data augmentation can effectively increase the sample size [39]. Inspired by this, we performed the operations shown in Fig 3(C), including horizontal flip, vertical flip, and random rotation. After these steps, the number of images containing SDTs was expanded to 2031. The software Labelme was subsequently used to label the 3940 SDTs in the expanded images, generating an SDTs label file in JSON format. The label was divided into three parts: training (70%), validation (10%), and testing (20%). Finally, a complete data set of SDTs is generated, which includes two types: broadleaved and conifers, with only their health status marked. The data in the test set is not enhanced.

**Table 2:** The number of SDTs in the test area.

Site	Number of SDTs images	Number of Labels on SDTs
Test area 1	76	131
Test area 2	104	179
Test area 3	47	81
Test area 4	341	587
Total	568	978

## 3. Methods

**Fig 4:**
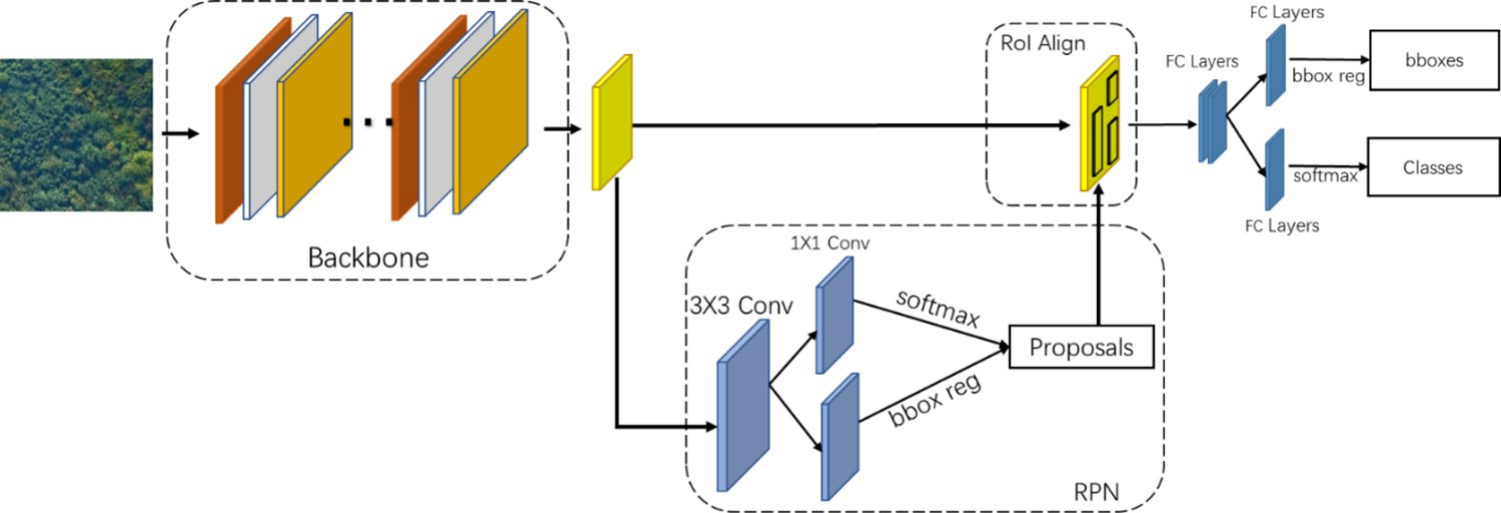
The architecture of faster-RCNN.

Object detection algorithms in deep learning have developed rapidly because they can extract abstract features from raw data and achieve high generalization. Since our study adopts the orthophotos processed by aerial photography for the detection of SDTs, more attention is paid to recognition accuracy. Comparatively speaking, two-stage algorithms are more suitable for SDTs detection than single-stage ones. so Faster-RCNN [40], a two-stage algorithm proposed by Ross B. Girshick in 2016 is used as the basic research framework. As shown in Fig 4, Faster-RCNN consists of an input layer, a feature extraction layer, a region proposal network (RPN), an ROI Align layer, and a fully connected layer.

### 3.1. Proposed methods

**Fig 5:**
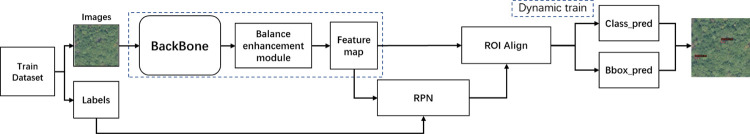
The overall flow chart of our proposed algorithm.

As UAVs can take photos at a high altitude, the scales of SDTs in the visible light images obtained by the UAV vary greatly. During the continuous process of feature learning, although the ability of object detection algorithms to express abstract features is enhanced, the spatial information in the shallow layer can get lost. Especially, because of the down-sampling operation, the deep network cannot obtain fine-grained spatial information, which will have a greater impact on small-sized SDTs in remote sensing images, resulting in a high rate of missed detections. In addition, small-scale SDTs are susceptible to the influence of surrounding discolored trees because of the similarity between the dehydrated canopy and discolored trees, and this similarity further increases the difficulty of identifying SDTs. To better solve the problems of image scales and identification difficulties. Our study integrates Swin-Transformer [41] and the FPN structure, and proposes a multi-scale feature enhancement extraction model based on the attention mechanism. This structure uses the dual features of distinct texture differences and color differences between dead trees and surrounding objects to identify dead trees. This integration is inspired by attention mechanisms and feature pyramid networks [42]. Our improved model uses top-down and lateral connections while adding functional balance enhancement modules. Based on the global analysis, shallow spatial information and deep semantic information can be effectively extracted. As shown in Fig 5, this is the overall flow chart of our proposed algorithm.

### 3.2. Multi-scale feature extraction model

**Fig 6:**
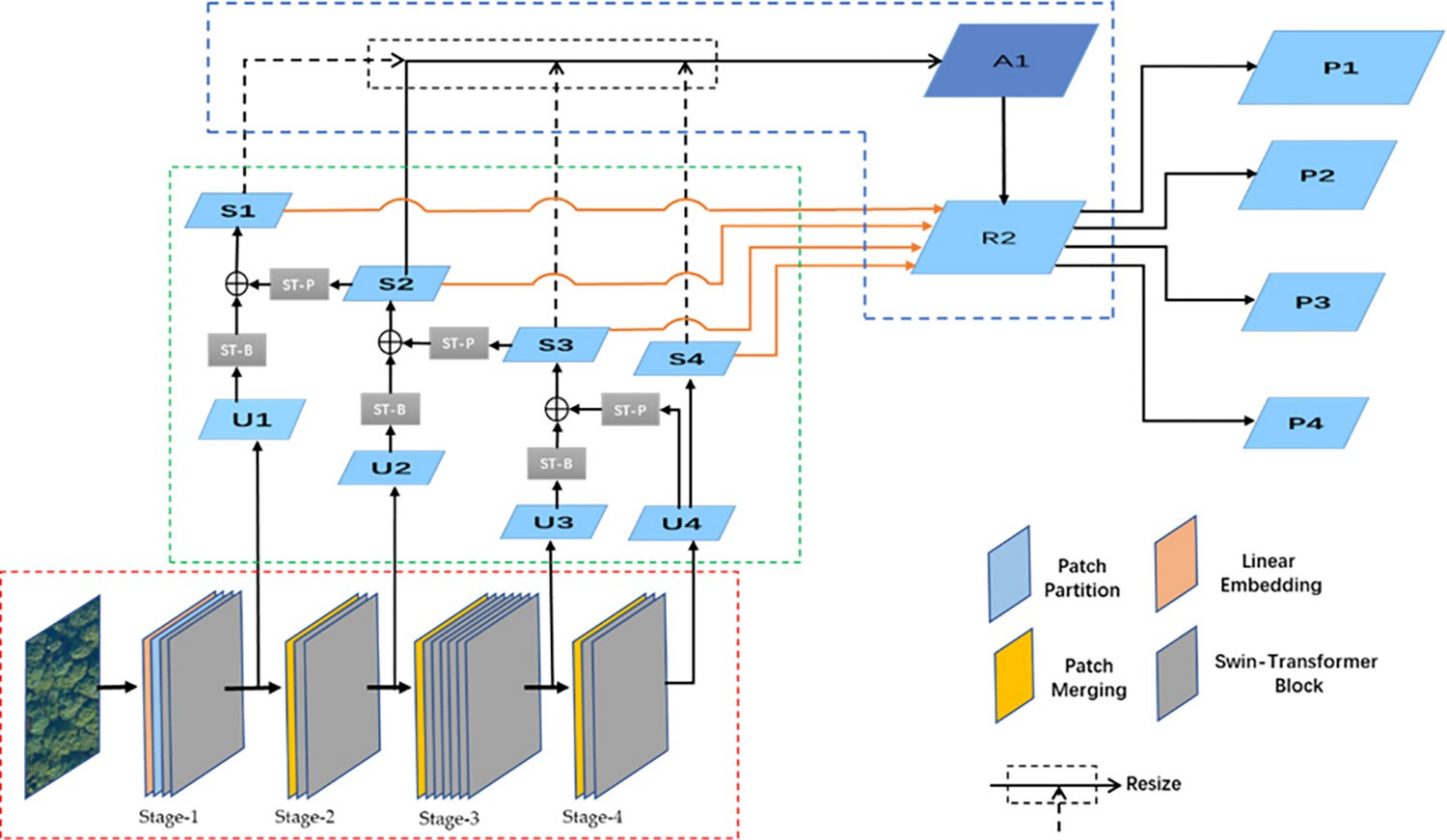
Structure of the proposed method.

**Fig 7:**
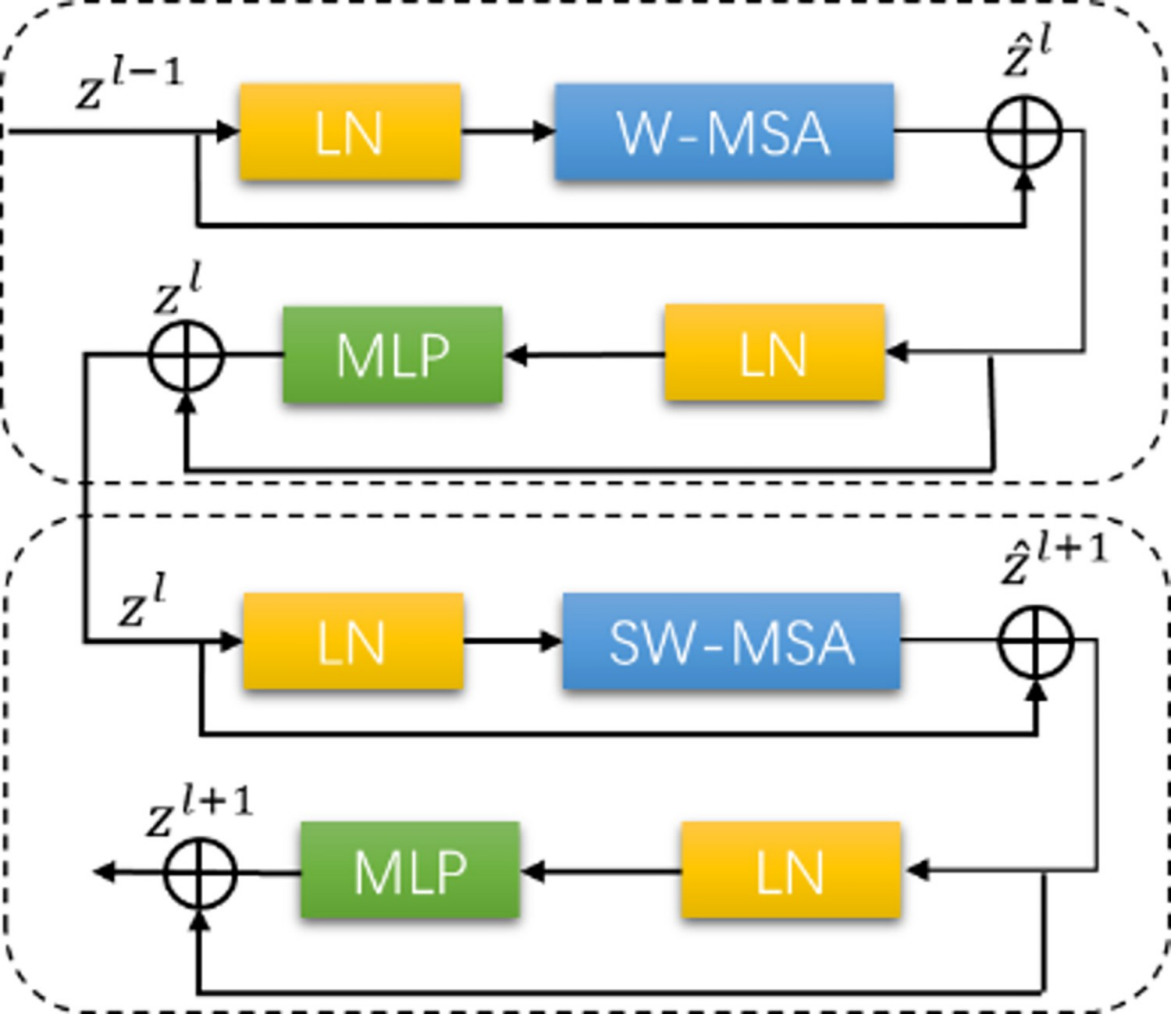
Swin-transformer block (ST-B).

As shown on the left side of Fig 6, this is our proposed Swin-Transformer of the FPN structure. This structure is based on the construction of global information, and then the features of SDTs are extracted using a multi-scale structure. There are contains two main operations, one is the ST-M operation which lowers resolution and increases the number of channels. Conversely, the other is the ST-P operation which increases resolution and reduces the number of channels. Both operations include the Swin-Transformer block (referred to as ST-B), as shown in Fig 7. It can be seen from the Figure that ST-B includes upper and lower types, which generally appear in pairs. The ST-M operation involves patch merging, which extracts and merges the features divided into patches to form a new patch of 2×2 size. and then use the Concat method to fuse. After fusion, it passes through the LayerNorm and Linear layers, the image resolution is reduced by half, and the feature dimension of the image doubles. The ST-P operation involves patch expanding, which refers to adding a Linear layer to the input features so that the feature dimension will double in number. Then the rearrangement operation is executed so that the feature resolution of the image is increased twice whilst the feature dimension is decreased to 1/4 of the input dimension.

Once the original image (HxWx3) is input into the model, the image will be first divided into non-overlapping patches through the Patch Partition operation. For the convenience of calculation, the patch size is 4x4, and the image size is H/4×W/4×48. Subsequently, we perform three ST-M operations and after the operations, we perform a linear embedding operation to embed the location information that can be used for learning. As can be seen from the number of gray boxes in Fig 6, the number of ST-B contained in the four stages is {2, 2, 6, 2}. After these four stages, a four-layer structure constituted by U1, U2, U3, and U4 will be generated, and the size of each layer is H/4×W/4×C, H/8×W/8×2C, H/16×W/16×4C, H/32×W/32×8C respectively. Swin-Transformer involves four stages by default, in order to achieve a better detection effect, the FPN structure used in this paper also 4 stages. First, the layer of U4 has two output branches. One branch first outputs S4 by changing the number of channels through ST-B, and the other branch performs up-sampling through ST-P and integrates the obtained feature map with features from U3 after ST-B, as shown in Eq (1). In so doing, a feature output S3 is obtained. Meanwhile, S3 is up-sampled by the ST-P operation, which carries twice the ST-B block, after completing up-sampling and fusing features with U2 after the ST-B operation to generate S2. In a similar way, a newly generated S2 is fused with U1 to generate S1. Once such operations are completed in sequence, S1, S2, S3, and S4 will be generated.


$$Z_{add}=\sum_{i=1}^{C}\left(X_{i}+Y_{i}\right)*K_{i}$$ (1)

In this formula, * denotes the ST-M.

**Swin-Transformer Block:** Swin-Transformer block consists of LayerNorm (LN), a multi-head attention module, a residual connection, and a MultiLayer Perceptron (MLP) with GELU nonlinearity. There are two kinds of attention modules. One module is a Window-based Multi-head Self-Attention module (W-MSA). Each calculation is performed individually on non-overlapping windows so that the computational complexity is linearly related to the image size.

The computational complexity of W-MSA is as follows:

$$\Omega (W-MSA)=4hwC^{2}+2M^{2}hwC$$ (2)

In this formula, h, w, and C are the height, width, and depth of an image respectively, and M is the size of the W-MSA window module.

The other module is the Shifted Window-based Multi-head Self-Attention (SW-MSA), which solves the problem that WSA lacks global information as a result of focusing on the information of a single window. The displacement is used to splice the information in the neighborhood so that the new window can obtain the unconnected information in the previous layer, thereby communicating information more effectively and obtaining global features. These two modules generally appear in pairs.

Relevant calculations in the Swin-Transformer block are as follows.


$${\hat{Z}}^{l}=W-MSA(LN(z^{l-1}))+z^{l-1}$$ (3)

$$Z^{l}=MLP(LN({\hat{z}}^{l}))+{\hat{z}}^{l}$$ (4)

$${\hat{Z}}^{l+1}=SW-MSA(LN(z^{l}))+z^{l}$$ (5)

$$Z^{l+1}=\mathrm{MLP}(LN({\hat{z}}^{l+1}))+{\hat{z}}^{l+1}$$ (6)

In the formulas above, ${\hat{z}}^{l}$ and *z*^*l*^ represent the output features of (S)W-MSA and MLP, respectively.

**Fig 8:**
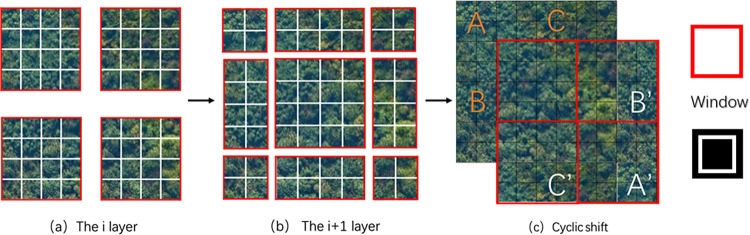
Window separation and calculation.

Fig 8(A) shows a picture divided into multiple regular small windows, on which W-MSA performs the self-attention calculation shown in Eq 3. Compared with the traditional Transformer, such an operation can reduce the amount of computation. Then, an image-to-image translation is performed and the area shown in Fig 8(A) is converted into nine windows, as shown in Fig 8(B) and 8(C) indicates image shifting, wherein the A, B, and C areas in the upper left corner are shifted to the position of A’, B’, and C’ in the lower right corner. This regenerates four regular windows and calculation is carried out immediately. Doing so enables cross-window connections to obtain more global information while reducing the amount of computation.

The self-attention calculation formula of Self-Attention is as follows:

$$Attention(Q,K,V)=SoftMax(\frac{QK^{T}}{\sqrt{d}}+B)V$$ (7)

Q, K, and V represent query, key, and value matrices, respectively. B is the relative position offset, and d represents the dimensions of Q or K.

### 3.3. Balance enhancement modules

In order to enable Swin-Transformer with a multi-scale feature pyramid structure to extract features of SDTs more effectively, this paper proposes using a feature enhancement module shown in Fig 5. The module has two parts, namely A1 and R2. A1 first combines the feature layers {U1, U2, U3, U3} output by Swin-Transformer with different layer features to get {S1, S2, S3, S4}. Then it uses bilinear interpolation and the maximum pooling to resize {S4, S3, S1} to that of S2 respectively and ultimately fuses the features of each layer by the following formula.


$$C=\frac{1}{L}\sum_{{l=l}_{min}}^{l_{max}}{C_{l}}_{}$$ (8)

In this formula, *l*_*max*_ and *l*_*min*_ represent features of the maximum resolution and the minimum resolution, respectively. *C*_*l*_ refers to the resolution features added to the L layer and there are L-level features in total.

**Fig 9:**
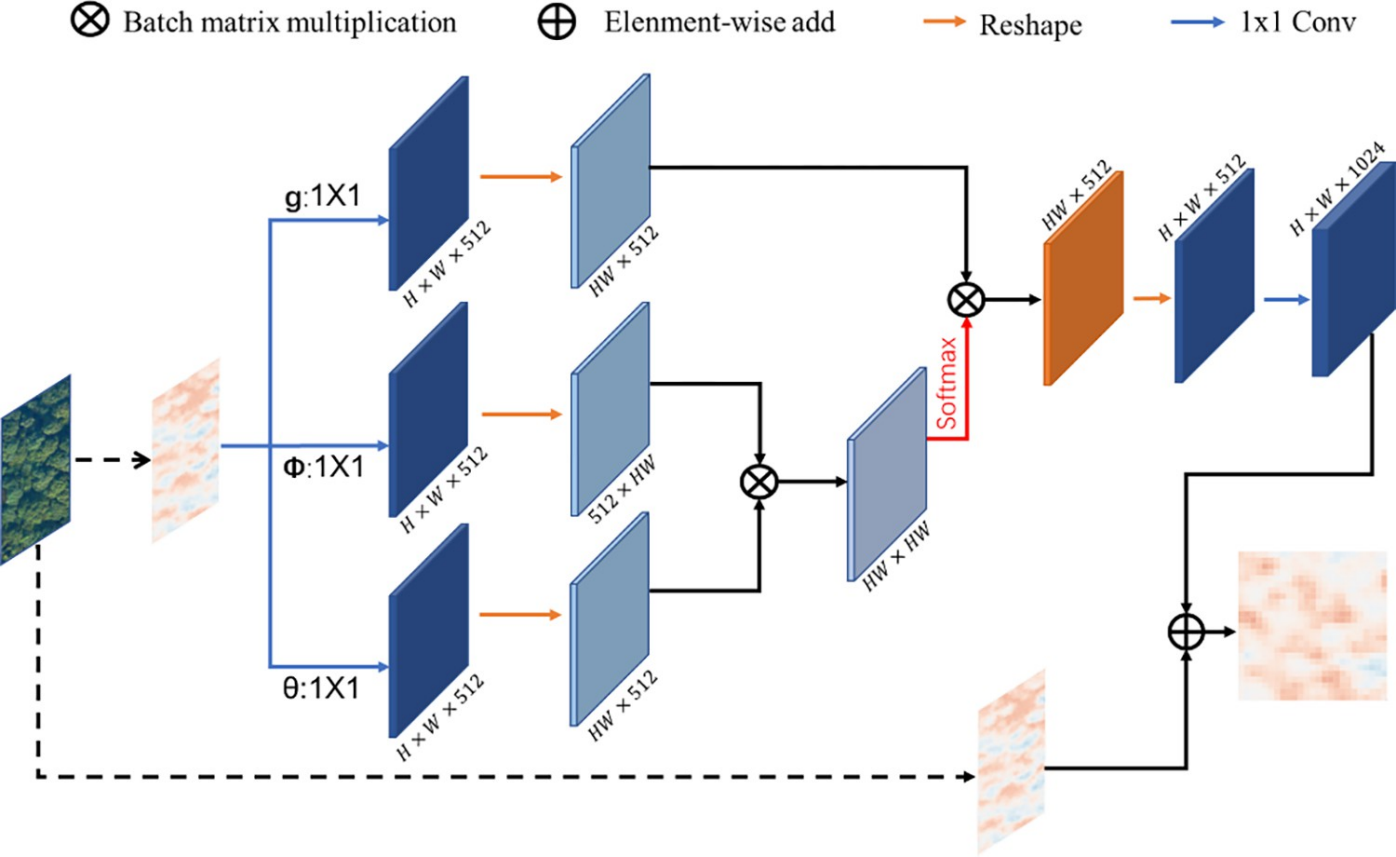
The embedded non-local block.

The feature layer after fusion in A1 will be sent to R2 for enhancement, hence balancing the fused information. In this process, the embedded Gaussian non-local attention [43] mechanism with the structure shown in Fig 9 is used to calculate the information between any two positions rather than only the information between adjacent positions by Eq 9. Therefore, the information on the fused features gets balanced, and more semantic information can be obtained. After the R2 process, the generated features are fused with {S1, S2, S3, S4} feature layers respectively to get the final feature layers {P1, P2, P3, P4}, which are finally sent to the RPN network for classification and regression.


$$Y_{i}=\frac{1}{C(x)}\sum_{\forall j}f(X_{i},\;Y_{j})g(X_{j})$$ (9)

Here, x is the input feature map, i is the index of a certain output position, j represents the index of enumerating all possible positions, $f(X_{i},\;Y_{j})=e^{{\theta (x_{i})}^{T}\varphi (x_{j})}$ is used to calculate the similarity between i and j, while *g*(*X*_*j*_) = *W*_*g*_*X*_*j*_ calculates the representation of the feature map at the j position. The final y is obtained by normalizing the response factor C(x) until its size is the same as that of the input x.

### 3.4 Dynamic training

In the training process of object detection, the method of Intersection Over Union (IOU) is used to measure whether the predicted boundaries match the real boundaries marked with labels. As shown in Eq 10, IOU means the calculated ratio of the intersection and union of the "predicted bounding box" (candidate bound, C) and the "true bounding box" (ground truth bound, G). An ideal situation is a complete overlap, that is, the ratio is 1. In general, the IOU threshold for distinguishing positive and negative samples is set to 0.5. It has been found that in the early training process [44], due to the small number of positive samples, if the threshold is set too high at this time, the performance of the model will be affected. Therefore, adjusting the threshold value with the change in the overall characteristics of the sample is beneficial to the performance improvement of the model. At the same time, with the different settings of the β value, the effect of the loss function of Smooth L1 will also be different. In order to improve the training performance, our improved method introduces a dynamic threshold and a dynamic loss function for training.


$$IOU=\frac{C\cap G}{C\cup G}$$ (10)

1. Dynamic IOU

The original label and threshold are shown in the formula below.


$$\mathrm{label}=\{\begin{array}{l} 1,\;\max IOU(b,G)\geq 0.5\\ 0,\;\max IOU(b,G)<0.5 \end{array}$$ (11)

In order to better train the model, dynamic thresholding is added. First, IOU is calculated on the generated candidate box and the actual label box, generating a series of IOU values. The first value *K*_*l*_ is selected as the current value. *T*_*now*_ in the formula below is updated every time the set number of iterations is reached. If there are multiple Epochs in one iteration, then the average of the previous *K*_*I*_ is chosen.


$$\mathrm{label}=\{\begin{array}{l} 1,\;\max IOU(b,G)\geq T_{now}\\ 0,\;\max IOU(b,G)<T_{now} \end{array}$$ (12)

In this formula, *T*_*now*_ represents the current IOU threshold.

1. Dynamic Loss

In order to make the model more stable, the Smooth L1 loss function is adopted, and the hyperparameter β is set to 1. The formula after setting is as follows,

$$F_{L}(x)=\{\begin{array}{c} 0.5{\left|x\right|}^{2},\;|x|<1\\ |x|-0.5,\;Otherwise \end{array}$$ (13)

Although original settings can help achieve better training quality, the fixed parameter setting cannot fit the data set properly. Thus, in order to make the model better match the SDTs data set, this paper replaces the loss function with F (x, β) of β which can update dynamically the data. The update method is similar to dynamic thresholding. First, the regression error is calculated based on the generated predicted box and the actual box. The regression error has four parameters. In order to reduce the computational complexity only the center coordinates and the average value, are taken and arranged in order. The Kth value is then selected, and once the set number of iterations is reached, the median is selected to update β_*now*_ in the following formula.


$$F_{L}(x,\beta )=\{\begin{array}{c} 0.5{\left|x\right|}^{2}/\beta_{now},\;|x|<\beta_{now}\\ |x|-0.5\beta_{now},\;Otherwise \end{array}$$ (14)

In this formula, β_*now*_ represents the median of the top K regression errors for this update.

## 4. Experiment and result

### 4.1 Experiment environment and details

Our experiment is implemented on the Ubuntu 16.04 system and the deep learning framework Pytorch1.8. Specific parameters are shown in Table 3. The pre-trained network model is imported through transfer learning to train the SDTs detection network. This helps detect SDTs in RGB images taken by UAVs. In terms of network training, the AdamW algorithm is used as the optimization algorithm, the initial learning rate is set to 0.0001, and Batchsize is set to 4. The learning rate is updated in a Linear way, and the number of iterations is 100. When the validation loss function does not change after 20 iterations, the training will be stopped in time.

**Table 3:** Software and hardware.

Name	Parameters
CPU	I7-10700K
GPU	NVIDIA GeForce RTX 3070
RAM	32GB
Operating System	Ubuntu 16.04
Frameworks and Languages	Pytorch 1.8 & Python 3.7
Operation Platform	CUDA11.2 & CUDNN V8.1.1

### 4.2 Evaluation metrics

During the experiment, the candidate box predicted by the model is compared with the real bound box annotated manually to evaluate the model. According to the matching degree calculated by IOU and the set threshold, the results can be classified into True Positive (TP), True Negative (TN), False Positive (FP), and False Negative (FN). Accuracy rate refers to the number of correctly detected SDTs to the total number of detections, and recall measures the percentage of actual SDTS that were correctly classified. The following are the formulas for Recall(R), Precision (P), Average Precision (AP), and F1-Score:

$$Recall=\frac{TP}{TP+FN}$$ (15)

$$\mathrm{Precision}=\frac{TP}{TP+FP}$$ (16)

$$F_{1}=2∙\frac{Precision\times Recall}{Recall+Precision}$$ (17)

$$\mathrm{mAP}=\sum_{k=1}^{N}P(k)\Delta r(k)=\int_{0}^{1}P(R)dR$$ (18)

P(R) represents the P-R curve.

### 4.3 Test results on the SDTs dataset

**Fig 10:**
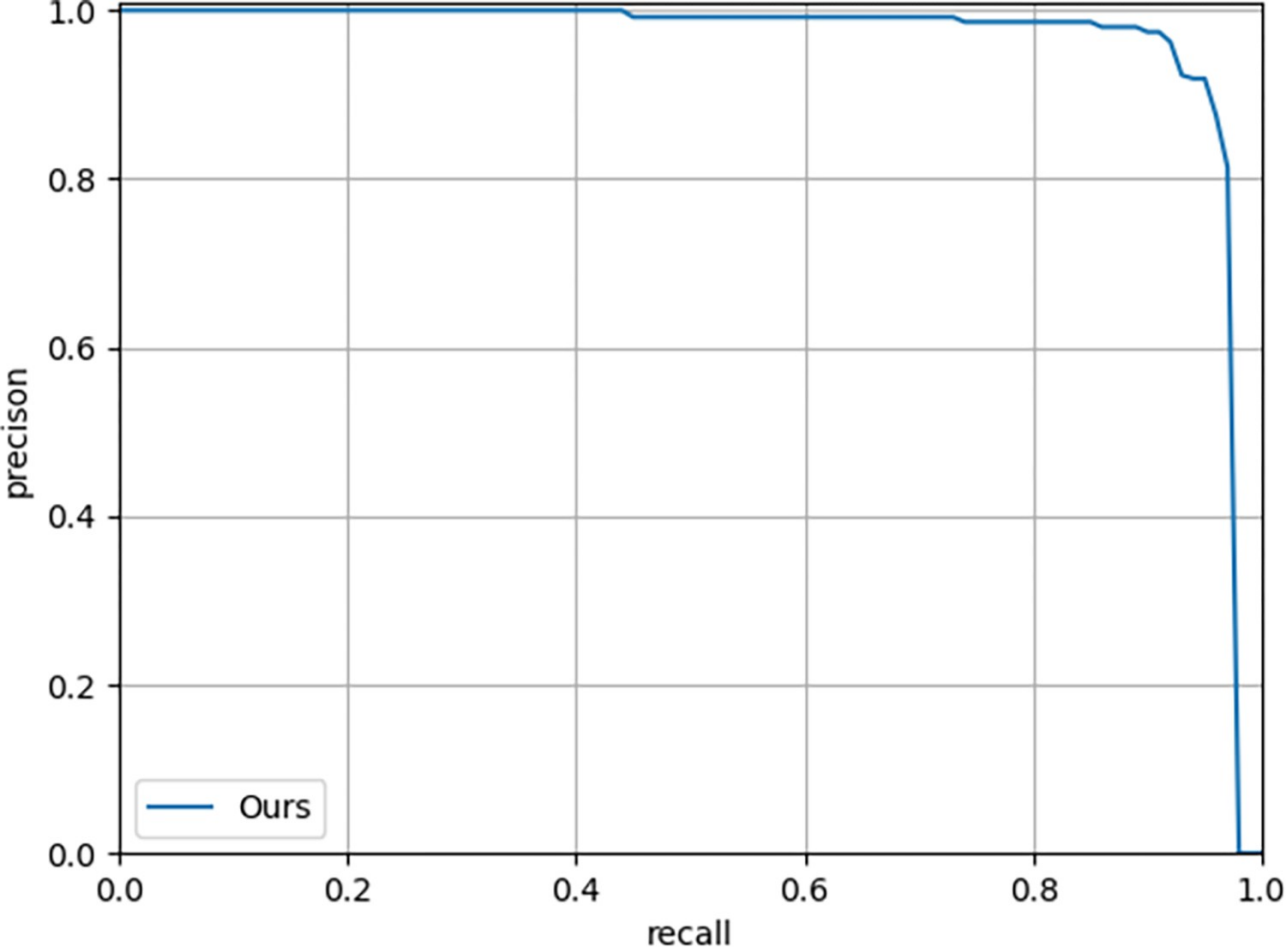
P-R curve.

**Fig 11:**
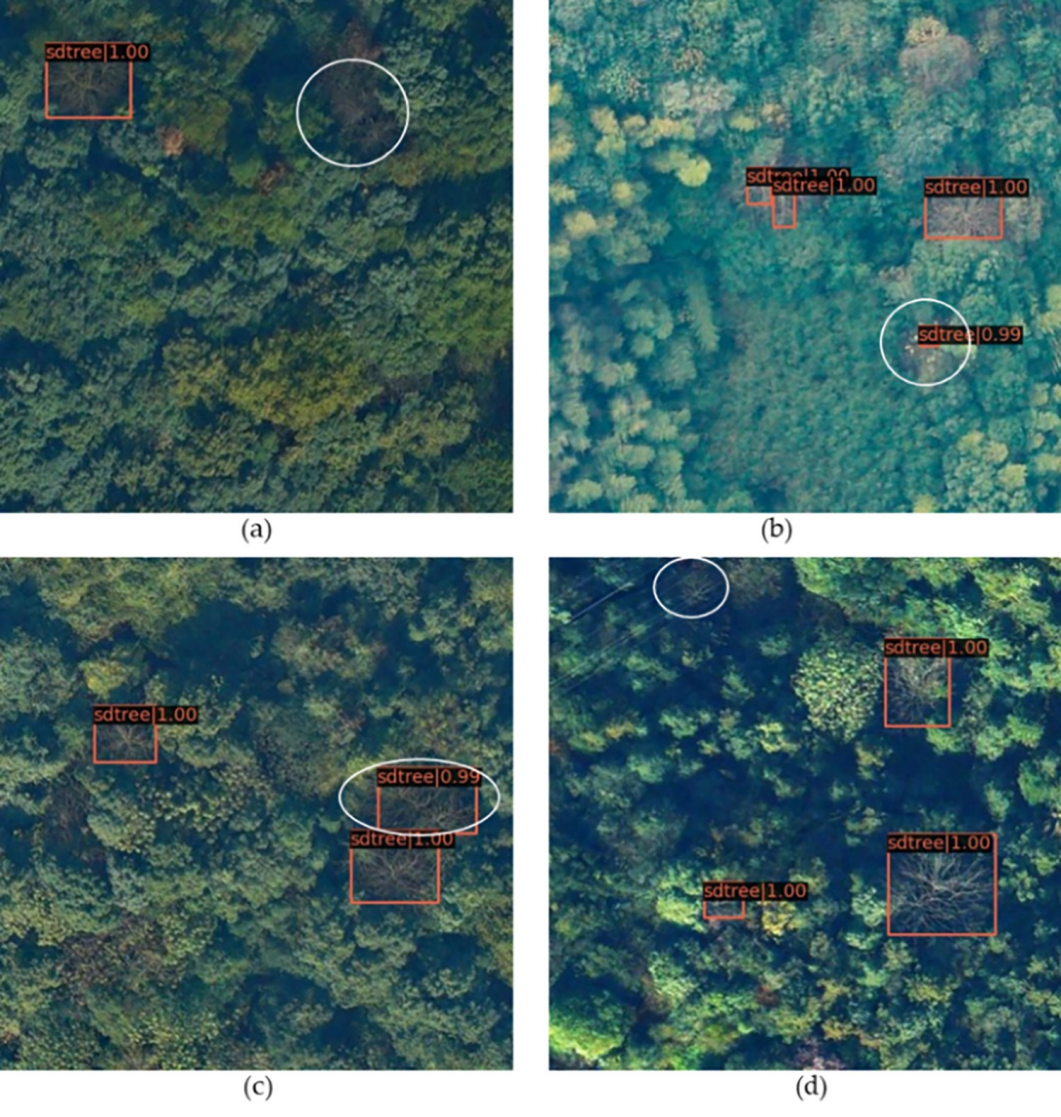
Errors in the improved algorithm.

In this paper, we use the SDTs dataset for training, and the type of dataset is COCO. We classify the picture whose pixel value is below 32x32 as a small-scale target, and that with a pixel value greater than 96x96 as a large-scale target. Data augmentation is only used for the training dataset, not used for the testing dataset. Based on training and testing on the SDTs data set, it is found that when the IOU threshold of the model is set to 0.5, the AP and Recall can reach 95.9% and 92.1%, respectively. The F1-Score is 93.9, which is calculated with reference to Precision and Recall. The number of images tested by this algorithm is 10.4 per second. Detailed experimental results are shown in Table 4. According to the Recall and Average precision, we draw the P-R curve as shown in Fig 10. The area enclosed by the P-R curve is the AP value, the larger the area, the larger the AP value, indicating that the model detection effect is better. During the testing process, the model also exists identification errors of SDTs, like misidentifications and missed identifications as shown in Fig 11. The white box indicates the place is problematic, and (a) is a missed identification, probably due to the poor texture features and low contrast of the two trees. (b) represents misidentifications, because the withered branches are similar to the loess in the forest, and the mark range is adjusted accordingly. (c) is a combination of missed identifications and misidentifications. One rectangular marker should have represented one marked-SDTs, but as seen from the Fig that two SDTs are marked together by one rectangular marker. This is a common problem in SDTs identification as canopies of SDTs in the forest are always overlapped or intersected. d) represents missed identifications, which can be caused by the same reason as (a).

**Table 4:** Test result.

mAP_50_	R_50_	mAPs	F1-score	FPS	Params/M
95.9	92.1	39.9	93.9	10.4	45.01

### 4.4 Ablation study

Ablation experiments are conducted to verify the influence of each module on the algorithm, as shown in Table 5. All experiment settings are the same. "Resnet" means the original Faster-RCNN, "Swin" means the Swin-Transformer with FPN, "B" represents the balance feature augmentation module, and "D" stands for dynamic training. Tick (“Π”) sign means that the module is used, while the absence of a Tick (“Π”) sign means that the module is not used.

**Table 5:** Ablation test results.

Resnet	Swin	B	D	AP_50_	APs
✓				88.9	-
	✓			93.9	35.1
	✓	✓		94.1	35.3
	✓		✓	92.3	35.0
	✓	✓	✓	95.9	39.9

As observed from Table 5, compared with Faster-RCNN whose backbone network is Resnet, Swin-Transformer, a multi-scale structure, is used in this paper to enhance the original backbone network. The AP is increased by nearly 7% overall and reaches 39.9% (0.5:0.95) on small objects, which is a significant improvement.

**Fig 12:**
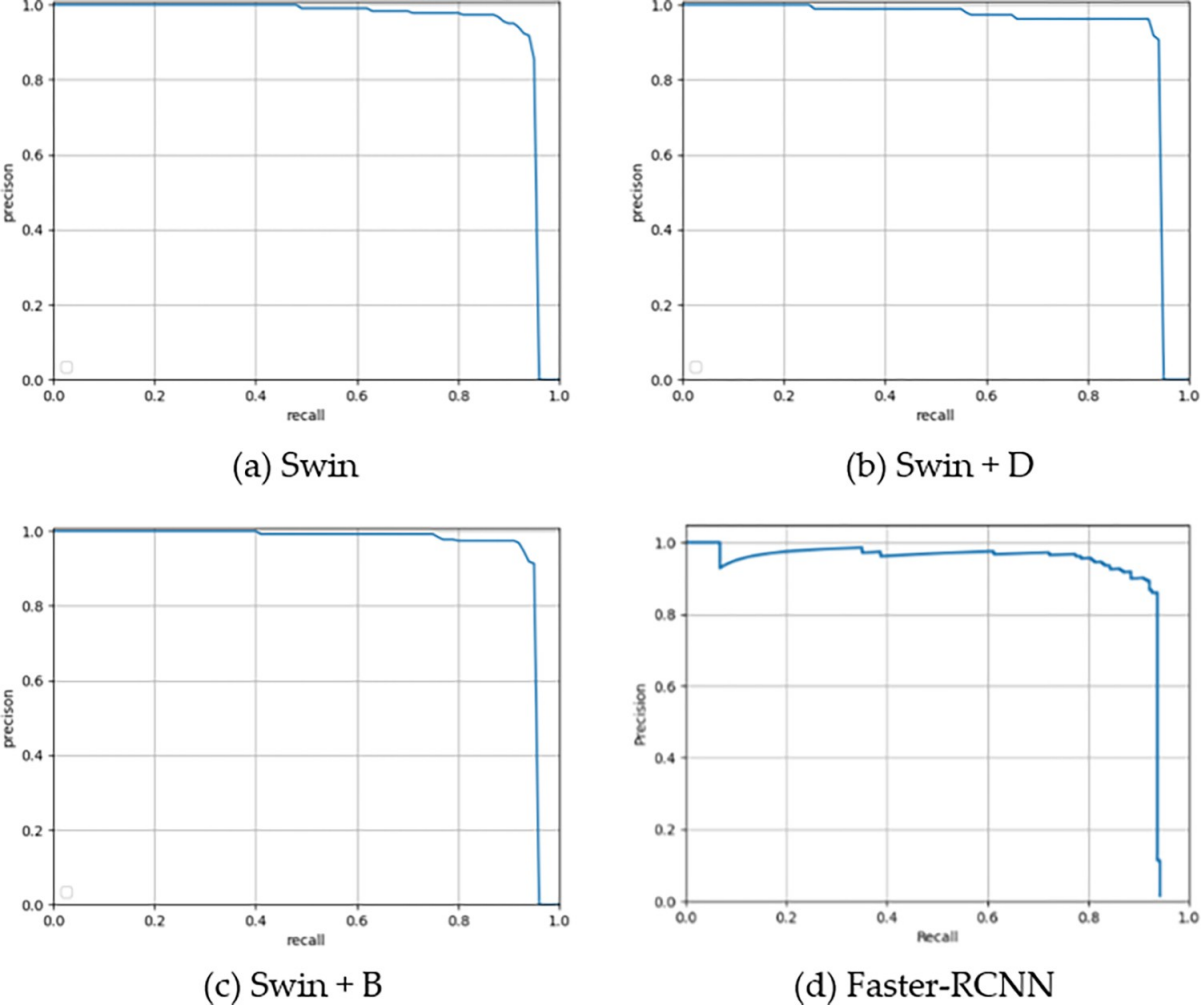
P-R curve for each part of the ablation study on the SDTs dataset.

In terms of improving the Swin-Transformer, after adding the multi-scale structure, AP reached 93.9%, and the AP of the small-scale target reaches 35.1%. Such an improved structure has shown a more significant improvement in the detection of SDTs. Furthermore, a balance feature enhancement module and a dynamic training model are added respectively. It can be observed that the AP (0.5) of the Swin-Transformer with only the balance enhancement module is increased by 0.2%, while the AP of the small-scale target is also increased by 0.2%, a small improvement. However, when only dynamic training is added, the AP (0.5) drops by 1.6%, and the AP of the small-scale target does not drop significantly. When both the Balance feature Enhancement module and the Dynamic Training module were added, the AP value increased by 2%, and the AP value of the small-scale target increased by 4.8%, respectively, the improvement is obvious. Fig 12 shows the P-R curve of each module, where (a) is the P-R curve with only multi-scale Swin-Transformer structure, (b) is the P-R curve with dynamic training added based on (a), (c) is the P-R curve with balance feature enhancement module added on the basis of (a), and (d) is the P-R curve of the original Faster-RCNN. Through these curves, the effectiveness of the improvement of these modules on the original Faster-RCNN can be visualized.

## 5. Discussion

### 5.1 Comparison of different methods on the SDTs dataset

As shown in Table 6, the results of using Grid-RCNN, RetinaNet, YOLO v3, DETR, the original Faster-RCNN, and our proposed method on the SDTs data set are compared and discussed. It is worth noting that YOLO V3 is a single-stage algorithm, and DETR is the first end-to-end algorithm combining Transformer with CNN. Each of the gradient descent algorithms used in the experiment is a Stochastic Gradient Descent (SGD). The learning rate is set to 0.0001, the linear method is used for attenuation, the Batchsize is set to 4, and the number of training sessions is 100 epochs. If the loss function does not change within 20 epochs, the training will be stopped and the test will be conducted. The algorithms used in comparative experiments have been debugged to their optimal state.

**Table 6:** Result of different methods on the SDTs dataset.

Methods	mAP_50_	R_50_	F1	FPS/S
**Grid-RCNN** [45]	94.6	90.3	92.4	12.1
**RetinaNet** [46]	93.0	85.1	88.9	13.9
**YOLO v3**[47]	89.5	85.3	87.3	**37.5**
**DETR** [48]	88.3	**92.4**	90.3	5.5
**Faster-RCNN**	88.7	90.1	89.4	10.8
**Improved Faster-RCNN(Ours)**	**95.9**	92.1	**93.9**	10.4

Table 6 shows that our proposed algorithm achieves the best accuracy and F1-Score, 1.3% and 1.5% higher than the second-best Grid-RCNN, respectively. However, the detection speed is slower, with the FPS being 10.4. Such a speed, the same as that of the original Faster-RCNN, is slower than that of YOLO v3 by 27.1. YOLO v3 is the fastest detection on the SDTs dataset. In addition, the Recall of DETR is up to 92.4, but the recognition speed is slow, and the FPS is only 5.5.

### 5.2 Detection effect of different methods

**Fig 13:**
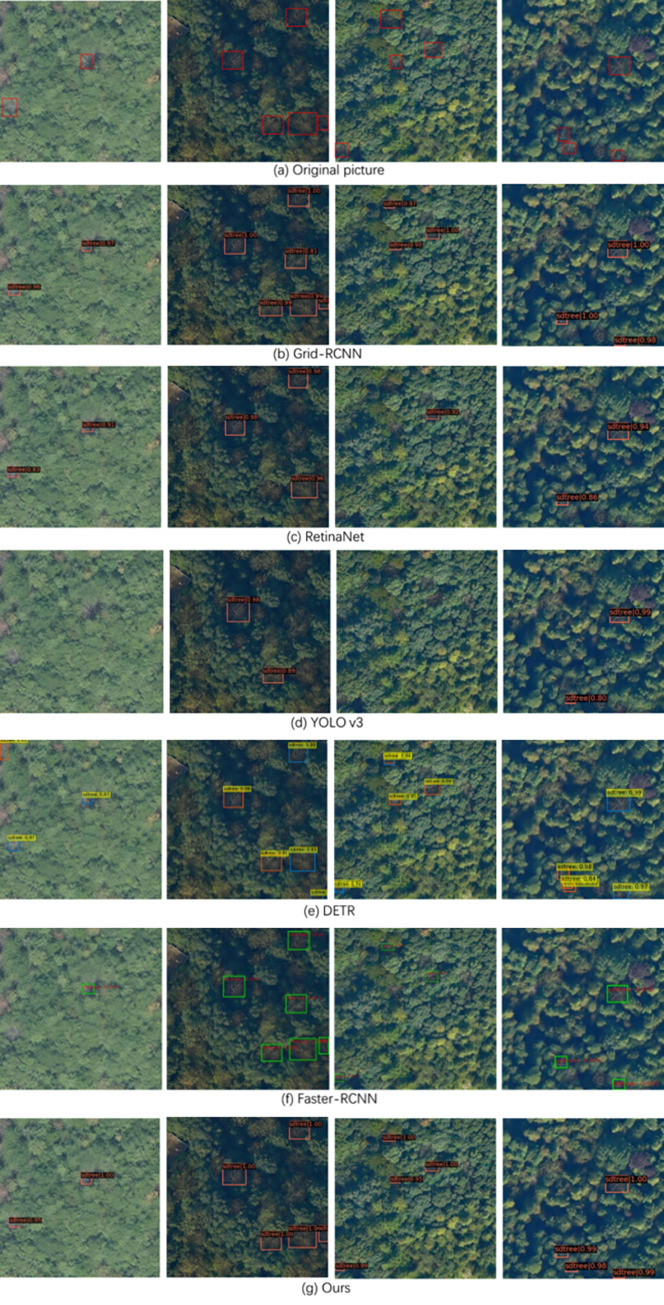
Detection effect of each algorithm. (a) Original pictures, (b) Grid-RCNN, (c) RetinaNet, (d) YOLO V3, (e) DETR, (f) Faster-RCNN, (g) Improved Faster-RCNN (ours).

Fig 13(A) shows the markers of SDTs present in the actual forest. It can be seen that tree leaves wither and fall off, and eventually only the crown remains. It is not easy to identify SDTs in structurally complex forests, and the problem is exacerbated by camera angle and lighting during the filming process. Fig 13(B)–13(G) present the identification and labeling of SDTs by different algorithms, the values in the upper right corner of each box indicate the confidence level of detection. Through the actual test, we found that among the comparison algorithms, YOLO v3 with Darknet as the backbone network performs the worst. Its F1-Score is 87.3, and there are many missed detections and misidentifications. Although YOLO v3 uses a multi-scale detection similar to the FPN structure, it is not effective in detecting small targets, despite a relatively fast detection speed. In a real-time environment, YOLO V3 has a certain effect on rough estimating SDTs in a certain area by using a UAV equipped with a digital camera.

Compared with YOLO V3, Grid-RCNN and RetinaNet adopt the feature pyramid structure for detection, and the detection accuracy for SDT is significantly improved. However, RetinaNet, as a single-stage algorithm, has a relatively low Recall which is at the same level as YOLOV3’s Recall. With a better recognition accuracy, the F1-score is 88.9 for RetinaNet, hence achieving a better overall effect than YOLO v3. Compared with the Faster-RCNN algorithm that uses regression to achieve proposal position correction, Grid RCNN adopts a position-sensitive, fully convolutional network to accurately position the target and achieve better detection. F1-score of Grid-RCNN reaches 92.4, and the Recall is basically the same as that of Faster-RCNN, which is 1.8% lower than our proposed algorithm in this paper.

DETR is the first end-to-end method integrating Transformer and CNN. The transform uses an attention mechanism, which can construct information globally and focus on hot spots locations in a way similar to human vision. In the detection of SDTs, the F1-Score of DETR can reach 90.3, and the Recall also has 92.4. The accuracy of DETR in identifying SDTs is slightly lower, and the convergence speed is slower than that of other algorithms. It can be observed from Fig 13(E) that there are many repeated identifications and misidentifications at the same position. DETR is also very computationally intensive in terms of data, the FPS is only 5.5.

Fig 13(G) shows the detection effect of our proposed algorithm. To be specific, the F1-Score can reach 93.9 in detecting SDTs, and the Recall has 92.1. Moreover, with the use of Swin-Transformer, which is characterized by moving window and grouping calculation, the FPS of our algorithm has been significantly improved, which is basically the same as Faster-RCNN and 4.9 higher than DETR. It can be observed from Fig 13 that regarding the same recognition task, the confidence score of our proposed algorithm is relatively higher than other algorithms. In particular, our improved algorithm has a better detection effect on the small-scale target and is more sensitive to the recognition of surrounding interfering objects.

Compared with previous algorithms, our algorithm has a significantly improved of recognition accuracy. However, since a few small-scale trees are well hidden in the forest, which still has missed identifications and inaccurate identifications during the detection process. For example, branches of adjacent SDTs are intermingled and thus misidentified as one tree. In other cases, branches of dead trees are mixed with living trees in the forest, these constitute obstacles to the identification.

## 6. Conclusion

Our study shows the potential of RGB images acquired by a low-cost UAV and deep learning to detect SDTs. We developed a forest SDTs detection algorithm based on the improved Faster-RCNN. The algorithm is mainly used to detect and mark SDTs from visible light images taken by UAV aerial photography. Due to the complex environment in the forest, in order to improve the overall detection accuracy of SDTs under complex conditions, the Swin-transformer based on global computing is used as the backbone network. Compared with the traditional transformer, the Swin-transformer has a smaller amount of computation. At the same time, in order to improve the detection accuracy of small-scale SDTs, this paper changes the Swin-Transformer in the feature extraction network to a pyramid structure and adds a feature balance enhancement module. By embedding Gaussian non-local attention to balance semantic information and spatial information, the ability of the network to extract multi-scale features, especially the features of small-scale objects, is improved. In addition, considering that the parameter settings of the different training periods will affect the training effect, dynamic training is introduced to dynamically adjust the parameters of the loss function and IOU threshold during the training process to make it more suitable for the SDTs dataset.

To verify the effectiveness of this algorithm, we conduct detailed experimental comparisons on the SDTs dataset. The experimental results show that the improved model in this paper is 7.2%, 2%, and 4.5% higher than the original Faster-RCNN in AP50, Recall, and F1, respectively. At the same time, in terms of detection effect, it also has certain advantages compared with other algorithms. In addition, it is hoped that the SDTs detection algorithm proposed in this paper can provide help for forest exploration and promote the application of deep learning in forest exploration tasks.
